# Mammalian cochlea as a physics guided evolution-optimized hearing sensor

**DOI:** 10.1038/srep12492

**Published:** 2015-07-28

**Authors:** Tom Lorimer, Florian Gomez, Ruedi Stoop

**Affiliations:** 1 Institute of Neuroinformatics and Institute of Computational Science, University of Zurich and ETH Zurich, Winterthurerstrasse 190, 8057 Zurich, Switzerland

## Abstract

Nonlinear physics plays an essential role in hearing. We demonstrate on a mesoscopic description level that during the evolutionary perfection of the hearing sensor, nonlinear physics led to the unique design of the cochlea observed in mammals, and that this design requests as a consequence the perception of pitch. Our insight challenges the view that mostly genetics is responsible for the uniformity of the construction of the mammalian hearing sensor. Our analysis also suggests that scaleable and non-scaleable arrangements of nonlinear sound detectors may be at the origin of the differences between hearing sensors in amniotic lineages.

Nature provided our planet with an abundance of species. The question of how this abundance comes about has intrigued humans since early in their existence. In his treatise ‘On the Origin of Species’, Charles Darwin set forth in 1859 for a scientific explanation^1^, anchoring it in the general principles of competition. Since then, research on evolution has focused mostly on the particular twists and turns the course of natural selection has taken, trying to understand what advantage a specific modification would have given to its bearer.

Despite the high dimensionality of the space that must underly this optimization process, we observe in a number of instances an apparent convergence towards certain building principles, which is puzzling. The mammalian ear is one of these examples. After a long tradition of research on evolutionary linkage^2^,^3^,^4^,^5^,^6^ and on physiological and genetical correspondences of species^7^,^8^,^9^,^10^,^11^,^12^,^13^, it was suggested that convergent evolution may have directed insect^14^, as well as jointly insect and mammalian, audition^15^. Hearing in both cases may be mediated by the same key genes^16^, which would indicate a close evolutionary relationship. In mammalian audition, the anion transporter family *prestin* is expressed, whereas audition is mediated in nonmammalian vertebrates and in insects by prestin-homologous proteins^17^. The chordotonal organ (e.g. in Johnston’s Organ of the mosquito or of *Drosophila*^13^, c.f. Fig. 1), provides the sensory basis of most insect hearing. Although seemingly very different at first view, the human cochlear hair cell that we will later centrally deal with, follows genetically closely the building principle of the chordotonal organs^18^. These observations seem to point at a joint early origin and parallel evolution of the hearing system.

While these genetical or physiological approaches have shed a fascinating light on how a major biological sense evolved and developed, they do not provide the arguments as to how this may have led to the sensory uniformity that we observe in particular within the mammalian family, despite evolutionary sensory specialization.

Here, we investigate what role physics principles must have had in this process, and we do this exclusively at the level of a mesoscopic description (in contrast to a micro-mechanical view that in the present context would be less insightful). On this level, we will exhibit how physical principles constrain the solution space of optimal hearing sensors in such a way that for large frequency bands and sharp resolution hearing, a convergence towards the blueprint realized in the mammalian cochlea is highly likely to occur. From this, we will suggest that the close genetical relationship observed in the construction of the hearing sensors, while of interest in itself, should not be seen as the main origin of the phenomenon.

## Small-power single frequency sensing

We start by positing that sounds around a characteristic frequency are often of particular interest to the animal world (the question how periodic behavior emerges from complex entities such as animals is old; if desired, the reader will find an appendix indicating our view regarding this issue). For spotting a predator, or a conspecific for reproduction, hearing a weak sound first among competitors is a substantial evolutionary advantage. In the simplest case, identifying one characteristic frequency will be important and might be sufficient. Insect hearing illustrates this at a fundamental level: The male mosquito *Aedes aegypti* performs ‘near-field’ hearing with a sensor that is tuned to the wingbeat frequency of females^19^.

For sound detection and perception, very faint input level sounds first need to be amplified actively^20^,^21^,^22^ (i.e., by using energy in addition to that contained in the arriving signal). Later processing of the information can then proceed at a fully developed signal level. A quite general and deep physical principle provides this mechanism as follows (how the mechanism is effectively implemented, e.g. whether on a molecular, mechanosensitive or electromotile level, is at this point of the discussion not of importance). Bifurcation theory developed in mathematics thirty years ago dealt with the fact that if in physical systems parameters are changed, occasionally the solutions emerging from such systems change their nature^23^. By varying a parameter across a certain value (the so-called bifurcation point), the nature of solution changes, in many cases by going from rest into an oscillatory state. Close to the bifurcation point, the natural solution loses its stability, and small perturbations develop in a hardly controlled manner, until after a time lapse that scales with the inverse of the distance to the bifurcation point, the system settles back onto its natural solution. The closer a system approaches instability introduced by the bifurcation, the more small inputs to the system are converted by the system into huge responses. In this way, systems close to bifurcations have been proposed to be used as active small-signal amplifiers^24^,^25^.

Two prominent bifurcations^23^ are generic candidates for the required bifurcation: a saddle-node (tangent) bifurcation (such as that leading from quiescence to regular spiking in the neuronal Morris-Lécar equations) or a Hopf bifurcation^26^ (as found in the Hodgkin-Huxley axon equations). While both bifurcations may serve as small-signal amplifiers, the particular bifurcation delivers a specific fingerprint onto the amplification law, which in the insect case considered below, as well in human hearing^27^,^28^,^29^ points at a Hopf bifurcation as the relevant process.

## Evidence of small-signal amplifiers in animal hearing

In the insect case, evidence for a Hopf bifurcation underlying the amplification process is obtained as follows. Generally, biological small-signal amplifying systems rest below the bifurcation point to oscillation. The bifurcation point may, however, even be crossed under certain conditions, which can be used to infer the deeper nature of the active amplification process below the bifurcation. In the example of the Drosophila antenna^30^, an injection of biochemical dimethyl sulfoxide (DMSO) leads to a crossing of the bifurcation point, from stirred antennal vibrations to self-sustained oscillations (‘SO’)^30^. From the observed velocity time series of the antenna oscillations (Fig. 2a), an underlying generalized van der Pol system (equation: see caption Fig. 2) could be identified that operates in the close vicinity of a Hopf bifurcation. For expressing the short-scale oscillations, a term *A*_0_ cos(2*π f t*) was included into the equation (*A*_0_ = 70 and *f* = 600 Hz). This term does not compromise the nature of the bifurcation and can be omitted for the following discussion. An enlightening understanding of the amplification dynamics can be provided by the behavior around zero displacement position *x* = 0, where the nonlinear damping term *Pn*(*x*) < 0 implies that energy is injected into the system, indicating active amplification (Fig. 2b). Around *x* = 0, the nonlinear restoring force *Pm*(*x*), together with its first and second derivatives, are relatively small. This implies that for small receiver displacements, virtually no restoring force is present. By means of the negative damping term, the system is thus easily driven out to large amplitudes. The comparison between data and obtained trajectories reveals the close correspondence between the data and the model. After having determined the system equations for the fully self-sustained oscillatory state, we follow the system on the way back to below the bifurcation point (Fig. 3). The recorded data compared to the figures obtained from scaling the two polynomials by two factors *μ*_*m*_, *μ*_*n*_, demonstrate, that by doing so, we closely follow the biological changes, where *μ*_*n*_ first lags somewhat behind *μ*_*m*_, but then takes the lead. At the bifurcation point, which is where the linear analysis reveals a Hopf bifurcation (inset), *μ*_*m*_ is still positive. Below, but close to the bifurcation point, where the antennal system usually operates, system-specific details are drowned out by the bifurcation properties. This implies that any such system, in particular Drosophila’s antenna equations, can be described in its essential features by the prototypical Hopf equation^26^.

Comparison to the mammalian hearing system reveals that, from a fundamental dynamical systems view, insects and mammals share the generic function principles of the sensor. In the mammalian case, the nonlinear amplification is by electromotile outer hair cells embedded mechanically into the basilar membrane, working in the vicinity of, but below, a Hopf bifurcation. If stimulated by a signal of frequency *ω* close to the Hopf system’s characteristic frequency *ω*_0_, the system would oscillate at *ω*, at a considerable amplitude. The response shown in Fig. 4 embraces all the required amplification properties of a small-signal amplifier. It is worth noting that these amplification profiles are of fundamental importance; we will show that their properties are preserved the whole way up the auditory pathway. From this, the main properties of the mammalian hearing sensor can be reproduced and understood (31,32, in particular the supplemental materials). The outer hair cells in today’s cochleae emerged very early in evolutionary history, before even the split of the stem reptiles from which the amniotes evolved, approximately 400 million years ago^33^. Why, how and under what conditions they came to form the final mammalian hearing sensor, the cochlea, and how this shaped the mammalian perception of sounds, are the main content of the next sections.

## Scalability of the many-frequency sensor design

**Figure 1:**
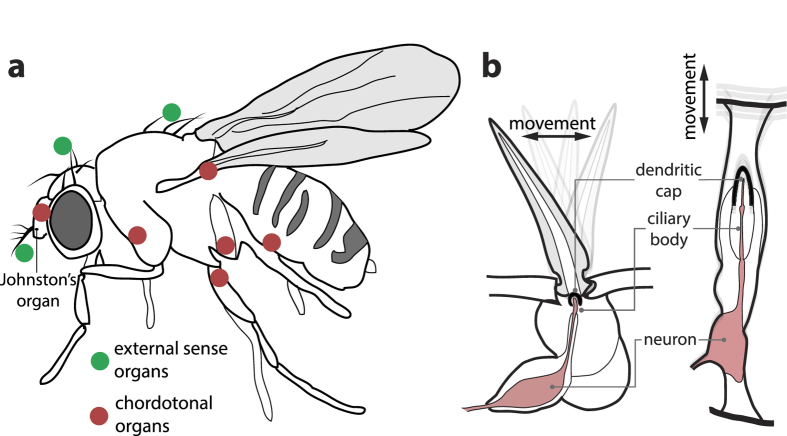
Sensory hair cells and chordotonal organs 18. (**a**) Locations of sensory hair cells (including the antennal receiver) and chordotonal organs in *Drosophila*. (**b**) Insect arista and chordotonal organs.

**Figure 2:**
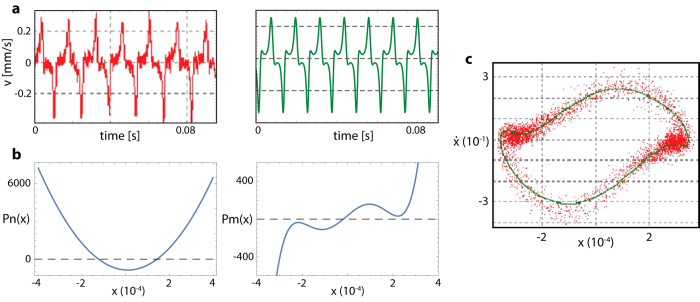
Fully developed self-sustained antenna oscillations (SO) of Drosophila, 20 min after DMSO injection (after Ref. 30). (**a**) Red: Data from^30^, green: simulations. (**b**) Best data-based polynomial approximating ordinary differential equation of SO 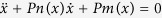, with polynomials of order *n* = 2 and *m* = 5, respectively. At extracted parameters, this system is close to a Hopf bifurcation, cf. Fig. 3. The damping term *Pn*(*x*) shows negative damping around the origin (*Pn*(*x*) < 0); the restoring force *Pm*(*x*) shows areas of negative stiffness *Pm*′(*x*) < 0. (**c**) Red: Data from^30^, green: low-passed reconstructed SO data.

**Figure 3:**
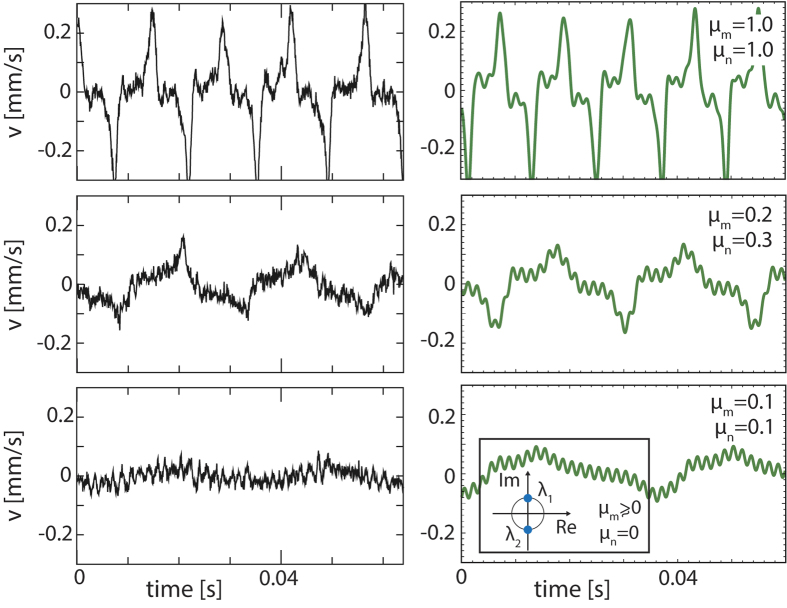
Top to bottom: From self-sustained antenna oscillations back to the quiescent fixed-point. Left column: experimental data from^30^, right column: simulation, where the polynomials were reduced by factors *μ*_*m*_ ≃ *μ*_*n*_. Close to bifurcation, *μ*_*n*_ precedes *μ*_*m*_, so that at bifurcation *μ*_*m*_ > 0. Inset: At crossing to quiescence, the linear analysis reveals a Hopf bifurcation^26^.

**Figure 4:**
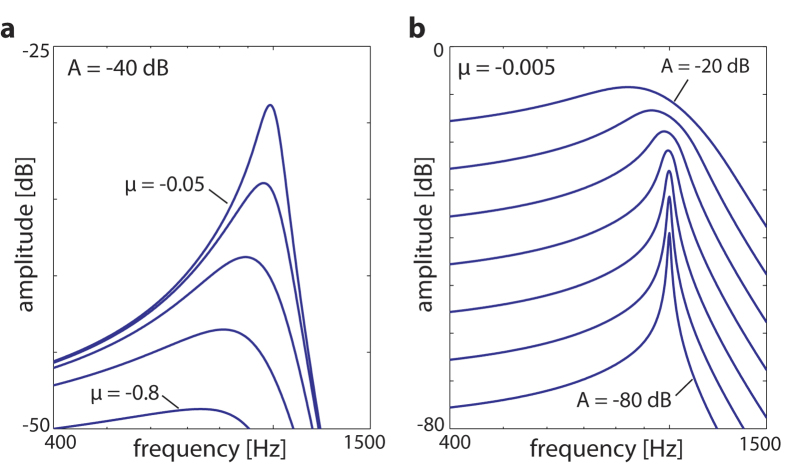
Single Hopf amplifier response^27^ conditioned on the passive behavior in the cochlea (leading to the asymmetry if compared to 24,25). The description mimics the behavior of outer hair cells with a preferred frequency CF embedded into the basilar membrane: Frequency selectivity (**a**) regarding different distances *μ* ∈ {−0.05,−0.1,−0.2,−0.4,−0.8} from bifurcation point, (**b**) regarding input signal strength (increase in steps of 10 dB).

The distinction of several frequencies, as emitted e.g. by a mate, predator, or prey, is of importance for survival and procreation in species that interact intricately with the world around. For a broad suite of frequencies, these hair cells must somehow embody a frequency tuning mechanism. The simplest solution on first view would be a construction by which each sensor inherently reacts to one specific frequency. In fact, the chordotonal organs and more specialized hearing organs that develop from them are found all over the insect body^13^ (Fig. 1). Because of the requested long wiring of such an arrangement, this concept is preferable only if relatively few frequencies are to be dealt with, as is naturally the case for small-sized animals, such as insects. Here is where the solutions taken by insects and mammals differ.

**Figure 5:**
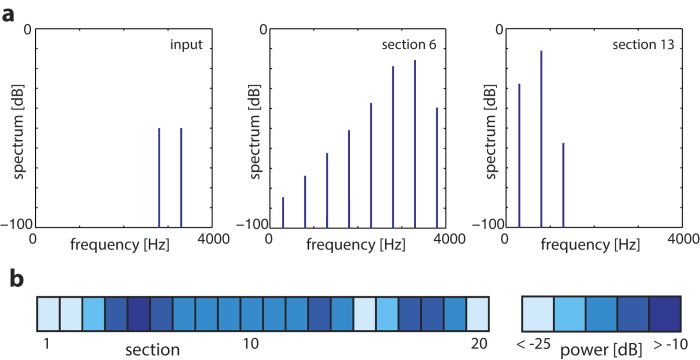
Patterns of evoked complexity in the ‘Hopf cochlea’ (45,50,51, for some details see the second-last section of the paper), generated by a simple two-tone stimulation. (**a**) Response (Fourier components) at the input, the 6th and the 13’th section of a discretized cochlea of 20 sections showing the emergence of combination-tone generated complexity down the cochlear duct. (**b**) Corresponding response profile (two-tone stimulation, −50 dB each tone, cochlea covering 7.04 − 0.22 kHz, all sections tuned to *μ* = −0.1).

For larger animals with an interest in a refined auditory environment, the natural solution is to locally concentrate the sensors. One complication, however, emerges: For nonlinear amplifiers, the superposition principle does not hold. Together with target frequencies, undesired interaction sound products are always generated (by amplifier interaction), which then are amplified by nearby amplifiers that have a characteristic frequency matching that of a combination tone (Fig. 5). Amniotes have such a locally concentrated solution, and live with the emergent complexity. The explanation of how they are able to cope with this challenge, will be postponed until the final section of this contribution.

**Figure 6:**
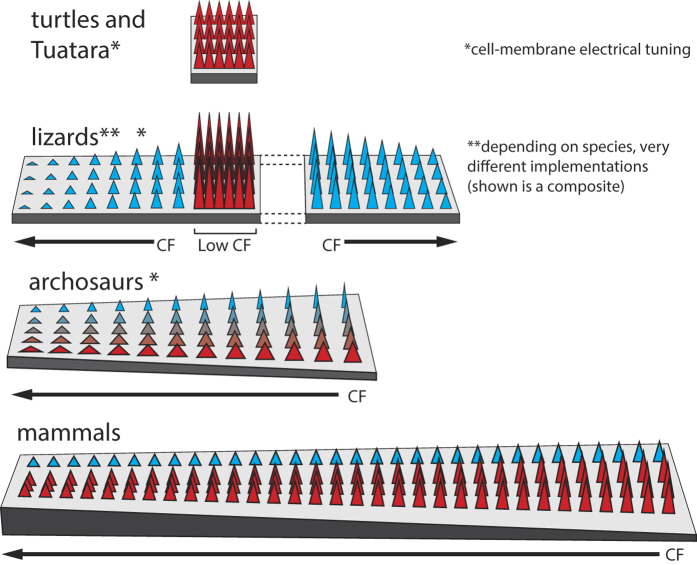
Schematic spatial arrangements and frequency tunings across stem reptile descendants, showing hair cell/bundle morphology (height/width), basilar membranes (as relevant to tuning), and hair cell innervation (blue: virtually none, red: increased efferent innervation). Hair cell membrane properties (electrical tuning), and orientation are not reflected. Short, unspecialized turtle and Tuatara basilar papilla are populated by a single type of electrically tuned hair cells. Lizard families separate high- and low-frequency areas on (modular) “untuned” basilar papilla, using different hair cell types. Mammals and archosaurs implement a single tonotopic gradient through basilar membrane stiffness and surface tension.

The simplest, relatively unsophisticated, arrangement of locally concentrated hearing sensors is found in the turtles and Tuatara, that probably still reflect the original stem reptile hearing system^5^ (Fig. 6 top). Their characteristic frequencies are electrically implemented; the range of their frequency sensitivity is generally very limited (below 1 kHz).

Lizards represent the next step of hearing sophistication. They show morphological gradients and variations of hair cells (Fig. 6, second row). Their frequency tuning is no longer purely electrical, but also of mechanical nature; the range of accessible frequencies in this family has noticeably enlarged^9^. Lizards have developed two distinct kinds of hair cells and separate them into type-specific areas, where only the low-frequency kind is provided with efferent connections that enable their neural frequency tuning^9^. This reduces the ‘listening’ capability of the sensor substantially^34^. Nonetheless, this construction already entails a substantial complexity of interaction signals, placing a significant cognitive burden higher up in their auditory pathway. Located half way toward hearing sensor sophistication shown in mammals, the great architectural variety that we observe could consistently be interpreted as locally optimized hearing solutions that are still at a distance from a global optimization solution. It may have been simply sufficient for lizards to minimize interaction products between sensors at a price of a much reduced hearing discrimination. Indeed, compared to mammals and birds, they base their living on auditory information to a lesser extent (they are largely non-vocal)^9^. The tokay gecko, which uses two types of hair cells similar in character to the mammalian inner and outer hair cells, may be seen as an exception^35^ that points into the direction of the next level of solution.

**Figure 7:**
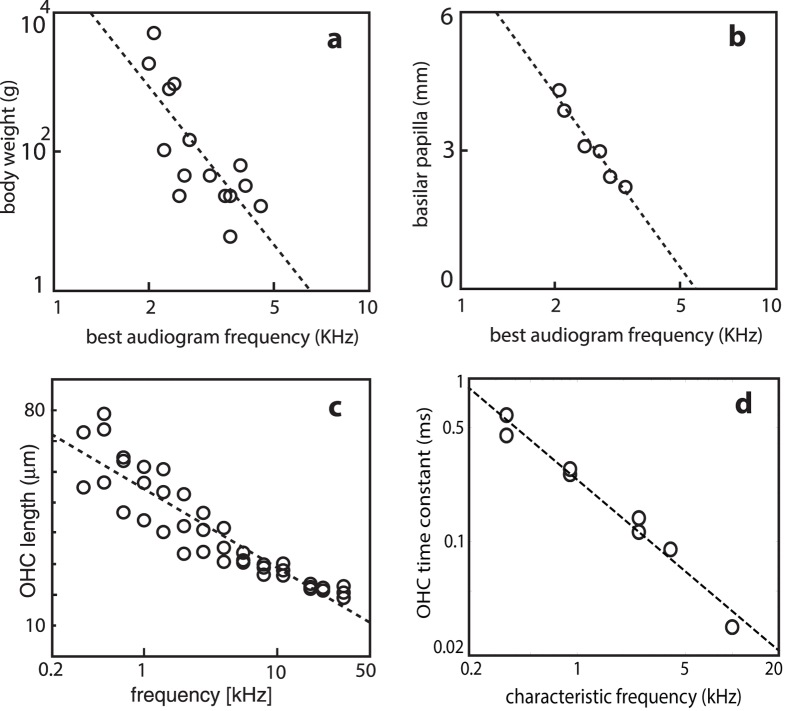
Scaling: (**a**) body weight, (**b**) basilar papilla length vs. best hearing frequency^40^; (**c**) outer hair cell length vs. characteristic frequency *in situ* (each circle refers to the mean from one guinea pig OHC row, based on data from Ref. 52), (**d**) outer hair cell time constant vs. characteristic frequency^53^.

For ultimately extended and selective frequency ranges, care needs to be taken in the overall construction scheme of the sensor. First, some broad structural arrangement of the characteristic (preferred) frequencies across the device is needed. The single one-dimensional frequency arrangement first observed in the archosaurs^36^ has clear advantages over other conceivable arrangements, as, by their definition, frequencies only require one dimension for discrimination (Fig. 6, third row). Some millions of years later than the archosaurs, mammals also adopted this solution (Fig. 6, fourth row). Both lines developed an elongated basilar papilla with two kinds of hair cells on it. Archosaurs still follow partially the evolutionarily older electrical tuning^5^, which is known to limit their high-frequency hearing^37^. This setback was only fully abandoned in mammals. As the most obvious parameters of mechanical frequency specification, hair-cell size might be seen, but membrane substrate stiffness and surface tension may be even more important. Indeed, investigations of the mammalian outer hair cells have revealed that a single hair cell is likely to be broadly tuned in isolation^38^; its sharp frequency specificity is mostly obtained from the embedding into the basilar membrane as the substrate. Exponential decrease of the basilar membrane stiffness and a corresponding modification of the surface tension along the cochlear duct^27^,^39^, establish in this sense a perfect ‘tonotopical’ collapse of frequency and distance space on a logarithmic scale. In bird and mammal hearing sensor construction, this may have led to scaling as their underlying construction plan. Scalability of the hearing sensor is important in the context of evolution of the species within a single family, where it is reflected in the emergence of approximate natural scaling laws between the properties of the originator of a sound and the sound itself. We observe, for instance that the relationship between the weight of an animal, and the frequency it hears best can be approximated by a power law (Fig. 7a). Moreover, the frequency of best hearing is correlated with the high-frequency limit of hearing: small species with a short basilar papilla hear higher frequencies, compared to larger species with a longer basilar papilla^40^ (Fig. 7b). The offered scalability would not have been perfect had it not been supported by the proper tuning of outer/inner hair cell by their size. As we go down the mammalian cochlear duct, to keep pace with the stiffness of the basilar membrane decaying exponentially, outer/inner hair cells increase their length (Fig. 7c), to entail compatible whole-cell slope conductances and capacitances^41^ (Fig. 7d). This concept has the advantage that the frequency properties of each sensor do not need to be genetically set, but follow essentially from the scaling of one single physical construction. Lacking a low upper limit in frequency space, mammals were pushed to an exquisite elongation of their basilar membrane, which then by spiraling for space, led to the mammalian cochlea’s final form. To us, these facts strongly point at a primary physical, in contrast to a genetical, origin of the convergence of the mammalian hearing sensor.

One of the driving organizational principles of Cortex is wiring optimization^42^. We have shown recently that an observed doubly fractal connectivity architecture of the cortex, minimizes the networks’ expenditure in terms of wiring length to achieve its computational functionality^43^. It seems not too far-stretched to ask whether the exhibited construction principles of the cochlea also serve a similar constraint regarding its interfacing with the cortex. Very stable scale-free avalanche size distributions of the excited localizations in the cochlea, in response to simple random stimulations, seem to hint into this direction, without providing, as yet, clear conclusive evidence (work in progress). Our thesis of physics guiding the evolution towards the cochlear hearing sensor, is, finally, corroborated by its incredibly uniform construction. The human cochlea, e.g., is extremely similar to that of a squirrel, cat, dog, or of a guinea pig. Given the general importance of hearing for mammals, we suppose that deviant construction plans would already have entered the scene, if preferable.

## How mammals deal with the evoked signal complexity

We now resume the discussion of how the mammals cope with the complexity (cf. Fig. 5) that is generated by the interaction of the nonlinear amplifiers, in exchange for an optimized construction scheme. To exhibit the existence and importance of the effect, let us quickly recall the main results obtained from a physics-rooted mesoscopic Hopf cochlea model, that are strongly corroborated by available biological laser-interferometry data. In a nutshell, the computational or hardware Hopf model of the cochlea is fully based on the properties of hearing that we have exhibited before: Small-signal amplifier on a basilar membrane in a fluid environment. For a detailed description of the model of the cochlea and our claim that it reproduces all salient phenomena of the biological cochlea (including, in particular, combination tones with the correct amplitudes, scaling and phase behavior), we have to refer to our Refs 31,32,44,45). Here, we reiterate that, the farther the wave elicited on the basilar membrane travels down the cochlear duct, the more the observed signal is dominated by successively generated combination tones^31^. This leads to unexpectedly complex excitation patterns observed along the mammalian cochlea even for simple input (Fig. 5).

**Figure 8:**
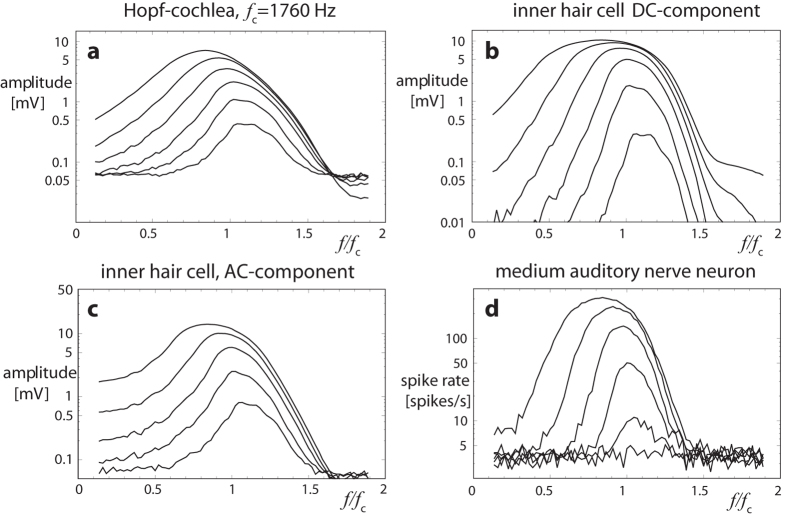
Mammalian hearing along the auditory pathway (**a**–**d**), at a chosen frequency ‘channel’. Vertical direction describes amplification characteristics, horizontal direction expresses frequency-tuning; lines refer to equal input levels. At the end of the pathway (**d**) the cochlear sound information is practically unchanged^32^ (analog cochlea implementation^45^,^50^,^51^). Despite substantial signal transductions occurring along this pathway, the original amplification profile, which is at the heart of the combination tone complexity, survives essentially unchanged.

From the classical signal processing dogma, undesired information should be filtered out as early as possible. Quite astonishingly, biological measurements and the corresponding models of the cochlea and cochlear nerve show that neither at the level of the cochlea nor higher up in the auditory pathway, does the mammalian auditory system make a noticeable effort to correct for the combination tones. Whereas filtering out at least some of the ‘artificial’ components seems perfectly possible for the biological system, this is just not how this system works^46^. Biologically detailed simulations of the auditory pathway indeed demonstrate that all the data collected at the cochlear level (including interaction products) are as faithfully as possible transported along the pathway, despite the astonishing variety of transformations and transductions they experience along this way (c.f. Fig. 8)^32^. This observation has even led to the insight that pitch is already present at the cochlear level and is not primarily a cortical product^31^.^32^,^44^. In fact, we explicitly showed^44^ that the pitch extracted from the continuous physics at cochlear level fully coincides with the pitch extracted at the end of the auditory nerve from discrete spikes^47^. The conclusion must be that physics as the claimed root of the convergence of the mammalian hearing sensor’s construction led to a unique perception of sound, taking account of all aspects of the signal’s evoked complexity.

**Figure 9:**
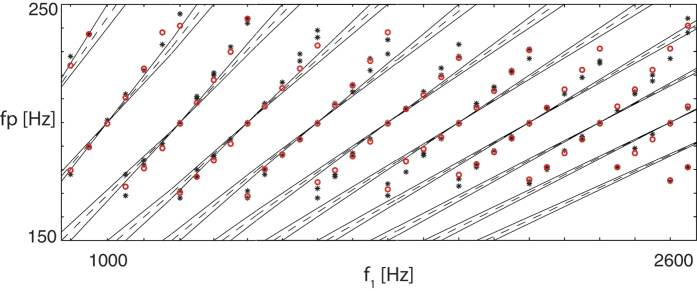
Perceived pitch *fp*. For two-tone stimuli (*f*_2_ = *f*_1_ + 200), Smoorenburg showed by his psychoacoustic experiments that the classically predicted pitch-shift of *δf*/(*k* + 1/2) does not emerge (‘second pitch shift’: black dots (partial sound levels 40 dB sound pressure level, two subjects) vs. black lines^48^). Red dots: Pitch extracted from a Hopf cochlea^44^.

From other fields of physics (e.g., how fractal dimensions or Lyapunov exponents describe the complexity generated by a chaotic process confined to a strange attractor), a common strategy for putting a grip on a complex phenomenon is to provide an overall ‘average’ characterization of the phenomenon. We now put forward that a similar effect could be the deeper nature of pitch perception. In the simple case of pure tone stimulations, pitch sensation coincides with the obvious physical properties of the stimulator. For slightly more complicated stimulations, the generated response develops, however, a profile of its own that departs substantially from the physical properties of the stimulating signal, due to characteristics that are rooted in the interaction among the nonlinear sensors. Such is the origin of the celebrated second pitch shift (Fig. 9) investigated by Smoorenburg. Motivated by the *missing fundamental* paradigm, Smoorenburg performed psycho-acoustical two-tone pitch-shift experiments. In these experiments^48^, the perceived pitch from an input of the form 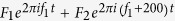 was evaluated by well-trained subjects, and compared to what the then known physical theories would predict. The human result was found to depart from what would have been expected from a ‘lowest order’ stipulated ‘fundamental frequency’ approach (first pitch shift phenomenon), and it also differed when the emergence of combination tones was taken (in a somewhat hand-waving way) into account (de Boers’s formula^49^, second pitch shift phenomenon). Moreover, the psychoacoustic experiments manifest up to three different perceived pitches for the same experiment.

To read out the pitch from our detailed model of the mammalian cochlea, we let Smoorenburg’s psychoacoustic and biophysical observations guide our precise measurement process, which implies that the perceived pitch *f*_*p*_ has to be computed from the dominant peaks of the signal’s autocorrelation function in the cochlea (for more details see Ref. 44). The obtained results are found to fully agree with the psychophysical evaluations^44^. In this work, the threefold pitch ambiguity was evidenced to be the coherent observation and the second pitch shift could be attributed to fluid-mediated sound wave transmission, an influence that previous theories of the perceived pitch had entirely disregarded.

## Conclusions

The observed construction convergence towards a uniform ‘mammalian’ cochlea thus appears as a natural consequence of nonlinear physics, rather than of genetics. The complexification of the auditory signal by amplifier nonlinearty, gives rise to the necessity of a ‘pitch sensation’ tool, needed to cope with the generated signal complexity, rendering a ‘purification’ of the compromised signal unnecessary. Very early in evolution, this might have been found to work much better than what classical signal processing methods could probably ever offer. Mammalian pitch sensation (as defined jointly in terms of physics and physiology in Ref. 44) permits the auditory system to identify or tag even an inharmonic sound by condensed information as a ‘fundamental frequency,’ even though the latter may be absent in the physical stimulus. This embracing property of pitch has recently been used as the main guiding principle for extracting desired elements of the auditory scene, which is at the heart of the cocktail party problem^34^ and corroborates the earlier claimed ability of the biological system to filter out undesired signal components, if needed.

Our evidence from fundamental nonlinear physics, supports and explains observed convergence in hearing sensor construction. It complements the physiological and genetical findings in a true sense, by reaching out towards the question why (instead of how) this happened. Combined approaches to the hearing system as an evolutionary prototype may finally shed light on the one fundamental question: What is the best computational framework for processing complex neural information? In light of the understanding that we have achieved regarding the first steps of the hearing pathway, such an expectation does not appear to be overly optimistic. If so, the physical principles underlying the hearing sensor evolution, would not only have provided us with (and have made us understand in a true sense) the blueprints for artificial hearing sensors of power and abilities that match the biological example, but would, moreover, reveal the fundamental principles followed by optimal signal processing.

## Additional Information

**How to cite this article**: Lorimer, T. *et al.* Mammalian cochlea as a physics guided evolution-optimized hearing sensor. *Sci. Rep.* **5**, 12492; doi: 10.1038/srep12492 (2015).
